# Diseases of Bones: Their Pathology, Diagnosis, and Treatment

**Published:** 1888-09

**Authors:** 


*Diseases of Bones: their Pathology, Diagnosis, and Treatment.* By THOMAS JONES, F.R.C.S. Pp. 369. London: SMITH, ELDER, & CO. 1887.

THIS work is an honest, original, and successful attempt to deal with the whole subject of bone-diseases. The author has personally worked at his subject; both clinically and pathologically his observations are mostly his own; and he has given due and discriminating attention to the work of others. The work is well and abundantly illustrated with chromo-lithographs, etchings, and wood-engravings; and most of the illustrations are original.

The book offers itself to criticism in respect of classification and arrangement. Thus, a chapter on Hypertrophy and Atrophy of Bone precedes the chapter on Inflammation of Bone. Inflammation of bone includes simple osteitis and periostitis, both somewhat scantily treated in one chapter of 20 pages; while osteomyelitis occupies two separate chapters, together occupying about 50 pages. This is probably a result of the special personal studies of the author. While we are very far from regretting the amount of consideration given to osteomyelitis, we should have liked to see the rest of the work written up to the same high level. In some minor matters, the same criticism might be applied. But we are loth even to seem to lower the high estimate we have formed of the work as a whole by carping at details. It is undoubtedly the best work on the subject which has recently appeared in these islands, and reflects great credit on the industry and research of the author.

